# A 1 bp deletion in *HACE1* causes ataxia in Norwegian elkhound, black

**DOI:** 10.1371/journal.pone.0261845

**Published:** 2022-01-21

**Authors:** Kim K. L. Bellamy, Fredrik S. Skedsmo, Josefin Hultman, Ellen F. Arnet, Ole Albert Guttersrud, Hege Kippenes Skogmo, Stein Istre Thoresen, Arild Espenes, Karin Hultin Jäderlund, Frode Lingaas

**Affiliations:** 1 The Norwegian Kennel Club, Oslo, Norway; 2 Department of Preclinical Sciences and Pathology, Faculty of Veterinary Medicine, Norwegian University of Life Sciences, Ås, Norway; 3 Department of Companion Animal Clinical Sciences, Faculty of Veterinary Medicine, Norwegian University of Life Sciences, Ås, Norway; University of Minnesota Duluth, UNITED STATES

## Abstract

A number of inherited ataxias is known in humans, with more than 250 loci implicated, most of which are included in human ataxia screening panels. Anecdotally, cases of ataxia in the Norwegian elkhound black have been known for the last 40 years. Affected puppies from three litters were clinically and neurologically examined, and postmortem samples were collected for morphological studies, including ultrastructural analyses. The puppies displayed vestibulocerebellar neurological signs and had degenerative histopathological alterations in cerebellum and brain stem. Three affected dogs, each from different litters, as well as both parents and one healthy littermate from each litter, were whole genome sequenced. Through variant calling we discovered a disease-associated 1 bp deletion in *HACE1* (CFA12), resulting in a frameshift at codon 333 and a premature stop codon at codon 366. The perfect association combined with the predicted significant molecular effect, strongly suggest that we have found the causative mutation for Norwegian elkhound black ataxia. We have identified a novel candidate gene for ataxia where dogs can serve as a spontaneous model for improved understanding of ataxia, also in human.

## Introduction

Ataxia is defined as lack of coordination of voluntary movements. Most often, ataxia is caused by lesions in the cerebellum, vestibular system, brain stem and/or spinal cord, but lesions in other regions may also be involved [1]. Several forms of hereditary ataxia have been described in both humans [2] and dogs [3].

Hereditary ataxias in humans may be classified based on their mode of inheritance: dominant, recessive, X-linked or mitochondrial. Autosomal dominant cerebellar ataxias typically have an adult onset and a slowly progressive disease course. The different types of autosomal dominant cerebellar ataxias (ADCAs) may be difficult to differentiate based on clinical signs. In addition to ataxia, clinical signs of ADCAs commonly include dysphagia, dysarthria, and signs of neuropathy [4].

Many ADCAs are caused by cytosine, adenine, and guanine (CAG) trinucleotide expansions. When CAG trinucleotides are located in coding regions they encode glutamine, which lead to the formation of polyglutamine tracts within the protein [5]. The affected proteins have little in common except for the polyglutamine, which indicate that the pathogenesis is directly linked to the long polyglutamine stretches [6]. It has been suggested that the polyglutamine may cause the formation of insoluble protein aggregates that accumulate in the neurons, resulting in cell death [7]. The pathogenesis of neurological disease caused by CAG trinucleotides located in non-coding regions is complex and largely undiscovered [8]. The length of the CAG trinucleotide expansions is negatively correlated with age of onset of disease. Some ADCAs are caused by other forms of expansions [5].

The autosomal recessive forms of hereditary ataxia are more diverse with regard to clinical signs and pathogenesis compared to ADCAs. The age of onset is often lower, usually with first appearance of clinical signs in early childhood. The exact pathogenesis is unknown for many of the recessive ataxias. However, some of the known mutations affect DNA-repair and/or mitochondrial function, or lead to the build-up of various macromolecules causing cell death [4].

The most common form of recessive ataxia in human is Friedreich ataxia (FRDA), which affects ≈ 1:30,000–50,000 of the population worldwide and is caused by mutations in the FXN-gene. FXN encodes the mitochondrial protein frataxin, which is involved in iron regulation. Most FRDA-cases are caused by GAA-repeat expansions. The age of onset is usually under 25 years of age. In addition to neurological signs, FRDA-patients also suffer from cardiomyopathy [9].

Other relatively common recessive ataxias include ataxia telangiectasia (A-T), ataxia with oculomotor apraxia type 1 (AOA1) and ataxia with oculomotor apraxia type 2 (AOA2), caused by mutations in the ATM-, APTX- and SETX-genes respectively, all of which encode proteins involved in DNA-repair. They are all progressive cerebellar ataxias, with various additional signs, including gradual loss of voluntary eye movements in AOA1 and AOA2. The first clinical signs are usually seen in early childhood [9].

Hereditary ataxias in dogs often have a recessive mode of inheritance [3]. The causative mutations are known in some breeds. Jenkins et al. recently showed that a SNP-variant in the KCNIP4-gene cause ataxia in Norwegian buhunds. The KCNIP4-gene encodes the potassium voltage-gated channel interacting protein 4, which modulates A-type potassium currents and is expressed in the canine cerebellum [10]. A GGA-repeat expansion in the ITPR1-gene, which encodes a signalling molecule receptor that is highly expressed in the cerebellum, is associated with spinocerebellar ataxia in the Italian spinone [11]. The ITPR1-gene is also associated with spinocerebellar ataxia in humans [12, 13]. In Alpine dachsbracke dogs, ataxia is caused by a mutation in the SCN8A-gene, which encodes a voltage-gated sodium channel and is also associated with ataxia in human [14]. Mutations in the KCNJ10-gene, which encodes a potassium channel, is associated with cases of heritable ataxia (spongy degeneration with cerebellar ataxia 1, SDCA1) in Belgian shepherds [15] and various terrier breeds [16, 17]. Another form of cerebellar ataxia in Belgian shepherds (SDCA2) is associated with a 227bp SINE insertion in the ATP1B2-gene [18].

Cases of ataxia in the Norwegian elkhound black (NEB) are described in breed club magazines, as early as in the 1980s. In 2014, the breed club was notified of a litter where some of the puppies displayed neurological signs suggestive of ataxia. In 2016, another litter was reported with similar clinical signs. The striking similarities between the affected puppies in the 2014- and 2016-litter evoked further investigations, which strongly suggested that the observed ataxia had a genetic background. Pedigree analysis revealed that the affected puppies from the 1980s, 2014 and 2016 have several common ancestors and were indicative of a recessive mode of inheritance. In this study, we present a thorough clinical and pathological characterisation of the affected dogs and report a new disease-associated mutation with a deleterious effect on HACE1.

## Results

### Pedigree analyses

**Fig 1:**
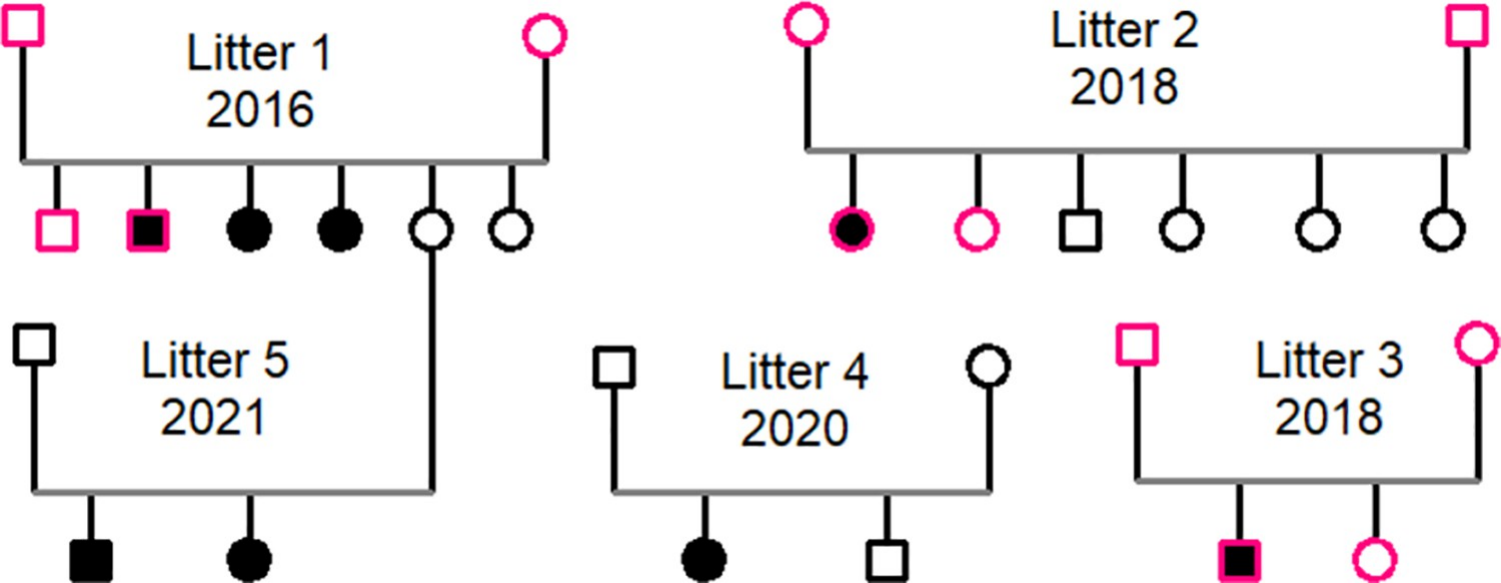
Pedigrees of litters 1–5. Sequenced (whole genome) animals in pink, affected individuals in black and unaffected individuals in white. Squares denote males and circles denote females.

Eight affected puppies were discovered in five litters (born 2016–2021) with a total of 18 offspring (Fig 1). All 10 parents were clinically healthy, and the segregation fit with an autosomal recessive inheritance.

### Clinical characterization of affected dogs

Owners reported an abnormal gait, especially in the pelvic limbs, of the affected dogs from around 4 weeks of age. The puppies were said to be unsteady, easily slipping on the floor with the pelvic limbs, occasionally falling over. In addition, they all had a hanging tail instead of the curled tail normal for this spitz breed. They all had from birth been alert with a normal appetite and a similar body weight as their healthy litter mates.

No abnormalities were observed on clinical examination, except for one puppy that disliked palpation of the pharyngeal/cranial cervical portion of the body and another puppy that behaved as in pain from extension/flexion of several joints in both thoracic and pelvic limbs and palpation of the spine. The puppies were all afebrile (median 38,5°C, range 38,1–38,7°C). On neurological examination, the body posture was kyphotic with a broad-based pelvic limb stance. The gait was moderately ataxic with hypermetric tendencies, most prominent in the pelvic limbs. Cranial nerves and spinal reflexes were unremarkable; however, the cutaneous trunci reflex could not be elicited in three of the puppies and in four of the puppies the menace reactions were absent (considered age-related). The different postural reactions tested were from mildly to severely delayed or absent in all four limbs of all puppies, with a slight asymmetry in three of the puppies. In conclusion, the neurological status suggested a vestibulocerebellar neurolocalization with a possible additional involvement of the spinal cord.

Hematology and serum chemistry results displayed no abnormalities, except for one dog with an increased C-reactive protein (CRP) concentration of 120 mg/L (normal upper reference limit: <15mg/L). This was the same dog that disliked palpation of the pharyngeal/cranial cervical portion of the body. Cerebrospinal fluid (CSF) analyses were unremarkable, as was the urine analyses. The computed tomography (CT) of the brain and spine pre and post intravenous contrast, was also unremarkable.

### Morphology

**Fig 2:**
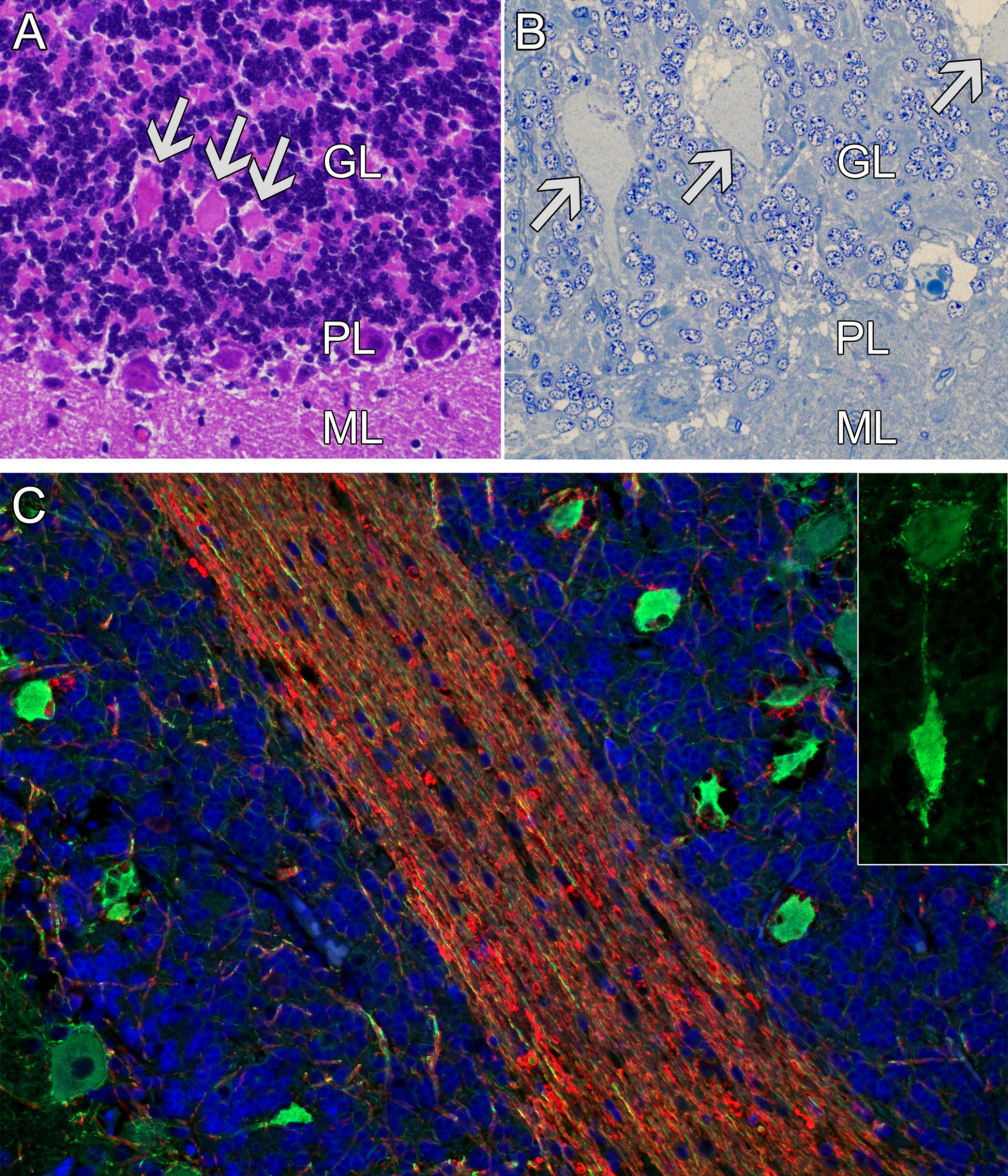
Cerebellar changes in affected dogs. (A) Light micrograph stained with hematoxylin and eosin. In the cerebellar cortex, homogenous eosinophilic structures (arrows) were present in the granular cell layer. (B) Light micrograph, semithin section stained with toluidine blue, showing the swollen structures (arrows) in the granule cell layer. (C) Immunofluorescence, antibodies against neurofilament (green) and myelin basic protein (red). Nuclei are stained with 4′,6-diamidino-2-phenylindole (DAPI) (blue). Swollen, neurofilament-positive structures were present in the granular layer, consistent with focal swellings in Purkinje cell axons (torpedoes). The connection between the Purkinje cell soma and swollen axon is displayed in the inset. GL = Granular cell layer, PL = Purkinje cell layer, ML = Molecular cell layer.

No macroscopical abnormalities were observed during the post-mortem examination. In the cerebellum of all the examined cases, homogenous eosinophilic axonal swellings were present multifocally in the cerebellar granule cell layer (Fig 2). These structures stained positively for neurofilament by immunohistochemistry and immunofluorescence and were consistent with spheroids in the Purkinje cell axons (torpedoes) (Fig 2). Occasionally, the torpedoes were shrunken and surrounded by a vacuolated space. In the brain stem, vacuoles were found in a moderate number disseminated and multifocally, both in the white matter tracts and in neuronal nuclei close to neurons. Degeneration of neuronal somas was not observed in any brain stem nuclei, including the olivary nuclei and the vestibular nuclei. No changes were observed in peripheral nerves, including the vestibulocochlear nerve, nor in sections from the spinal cord including dorsal root ganglia. No changes were found in corpus callosum and ventricles were not dilated.

**Fig 3:**
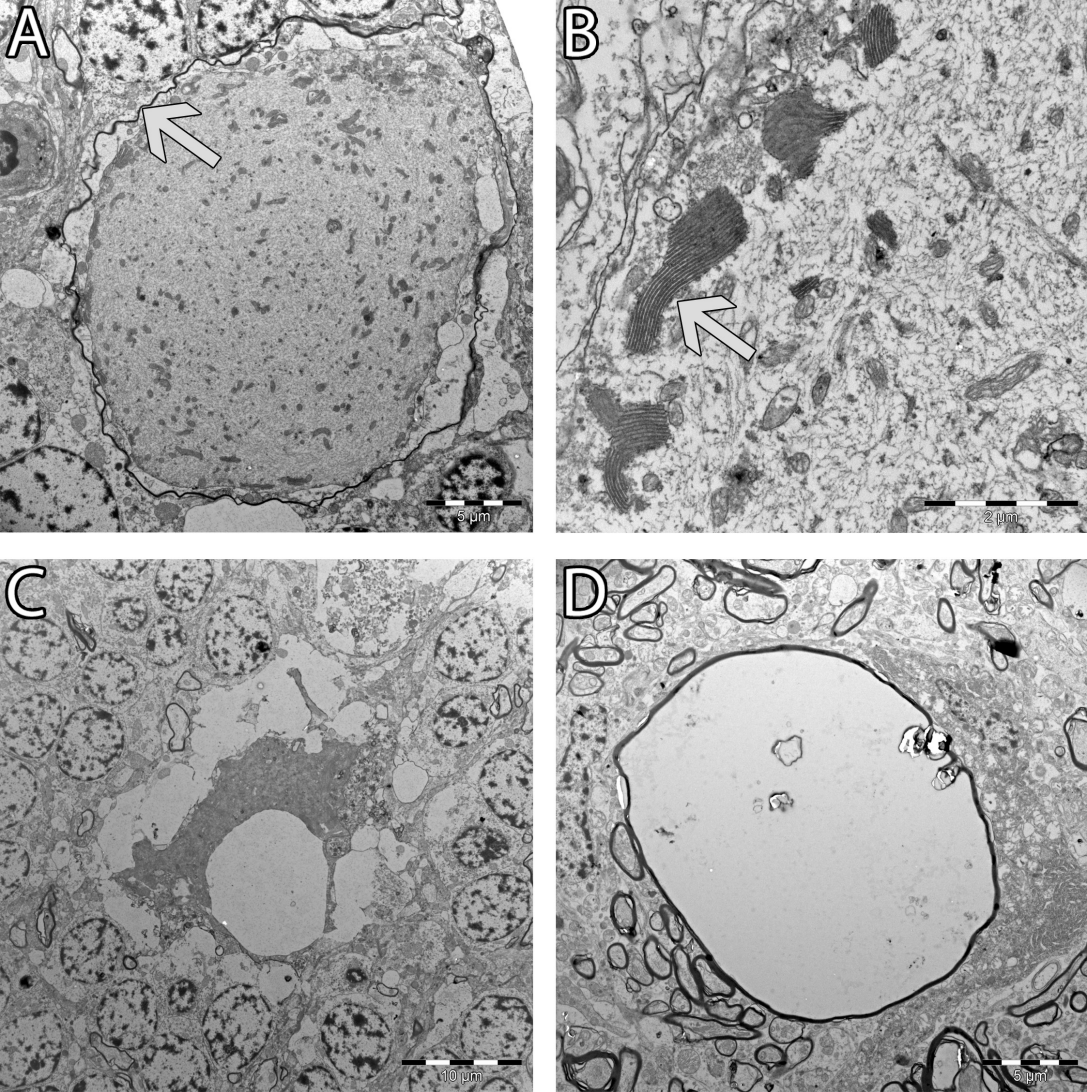
Transmission electron micrographs from cerebellum (A-C) and brain stem (D) of affected dogs. (A) The axoplasm of swollen Purkinje cell axons (torpedoes) were filled with disorganized neurofilaments and organelles. In some instances, they were surrounded by a thin myelin sheath (arrow). (B) Higher magnification of a torpedo. The organelles present in the axoplasm included mitochondria and membranous stacks (arrow). The latter were often found peripherally, close to the axolemma. (C) Some of the torpedoes were shrunken and surrounded by a vacuolated space. (D) In the brain stem, vacuoles surrounded by a thin myelin sheath were present.

Ultrastructurally, the content of the axonal swellings in the granule cell layers was dominated by randomly arranged aggregates of neurofilaments, mixed with mitochondria and membranous stacks (Fig 3). The stacks were consistent with smooth endoplasmic reticulum [19]. The mitochondria and membranous stacks were often found at the periphery of the axoplasm, close to the axolemma. In some instances, the torpedoes were surrounded by a thin myelin sheath, but most often no myelin sheath was observed. In the brain stem, vacuoles surrounded by a thin myelin sheath were present. The vacuoles contained a small amount of granular and membranous material, but in one instance an axon with myelin sheath was present in the periphery of the vacuole.

### Detection of a single bp deletion in *HACE1*

Whole genome sequencing and subsequent variant calling focusing on gene regions, selecting variants where parents were heterozygotes, affected offspring were homozygote variant, sequenced healthy littermates were not homozygous variant and three salukis were homozygote reference, detected only four closely linked private variants at CFA12 (Table 1). All four variants were located within *HACE1* (12: 62,227,078–62,337,183, reverse strand). Two of the variants (62229840 and 62230974) were located in the 5’-region and the other two (62282727 and 62282766) were located within the coding part of the gene. *HACE1* (HECT Domain And Ankyrin Repeat Containing E3 Ubiquitin Protein Ligase 1), contains 24 exons and encodes an E3 ubiquitin ligase.

**Table 1:** Four variants detected in *HACE1* (CFA12: 62,227,078-62,337,183 reverse strand).

CHROM	Position (CanFam3.1)	REF	ALT	Position
12	62229840	G	A	5’region
12	62230974	A	T	5’region
12	62282727	G	A	Exon 11
12	62282766	**AC**	**A**	Exon 11

**Fig 4:**
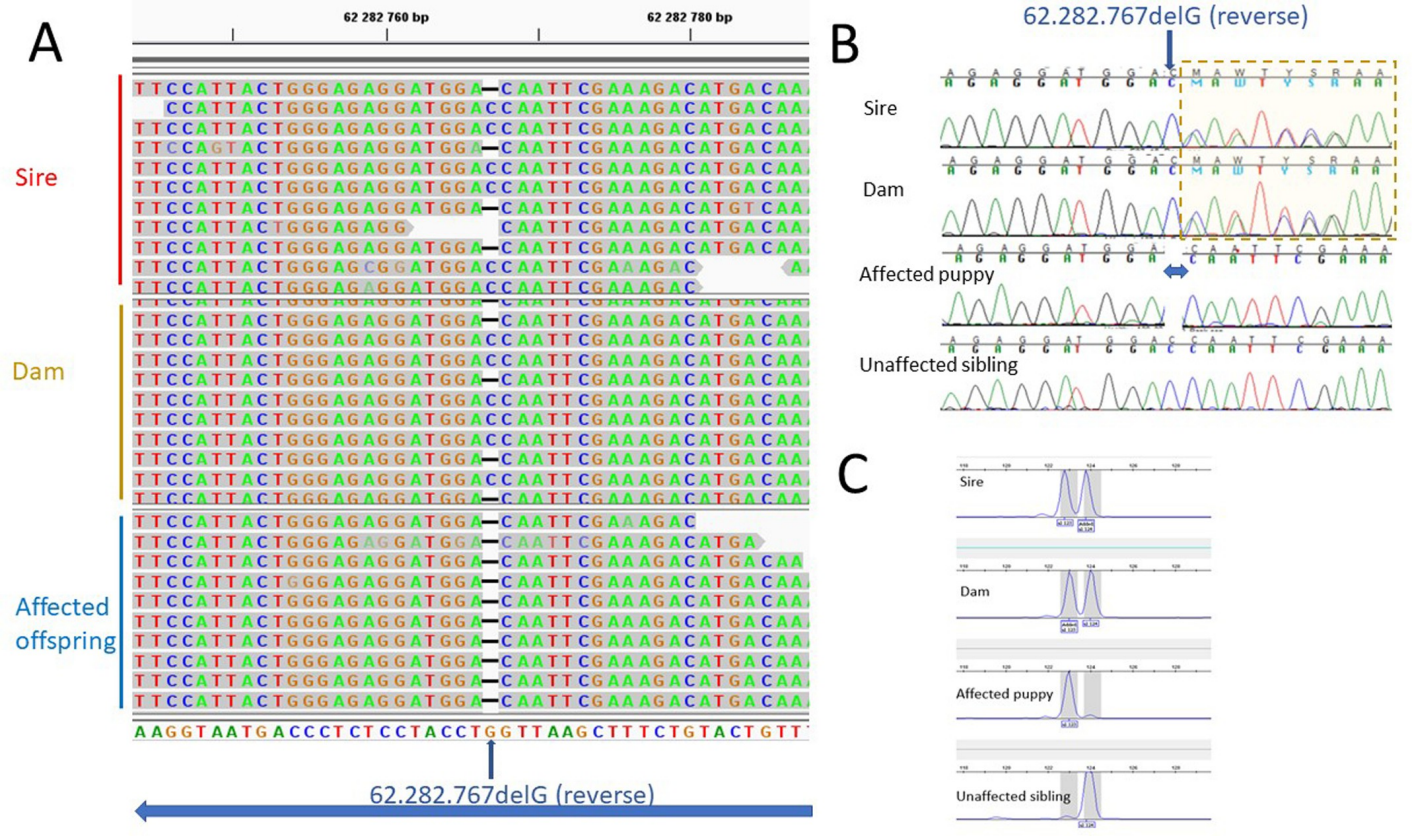
Sequences and fragment analysis. (A) WGS sequences of one family (25-35X) showing the deletion in exon 11 (illustrated in IGV). (B) Sanger control sequencing of heterozygote parents, affected puppy with homozygote deletion and healthy sibling (homozygote reference). Yellow square shows “double” Sanger sequence after deletion. (C) Fragment analysis of the same dogs as in “B”.

Both the *HACE1* variants (62282727 and 62282766) were located in exon 11 (ENSCAFT00000072236.1). 62282727 was an already annotated synonymous variant (AAC->AAT; N->N). The variant 62282766 was a 1 bp deletion (Fig 4) leading to a frameshift creating 33 new amino acids and a premature stop codon in exon 12. The original protein of 877 amino acids was reduced to 366 amino acids.

### Predicted effect on amino acid sequence and protein structure

**Fig 5:**
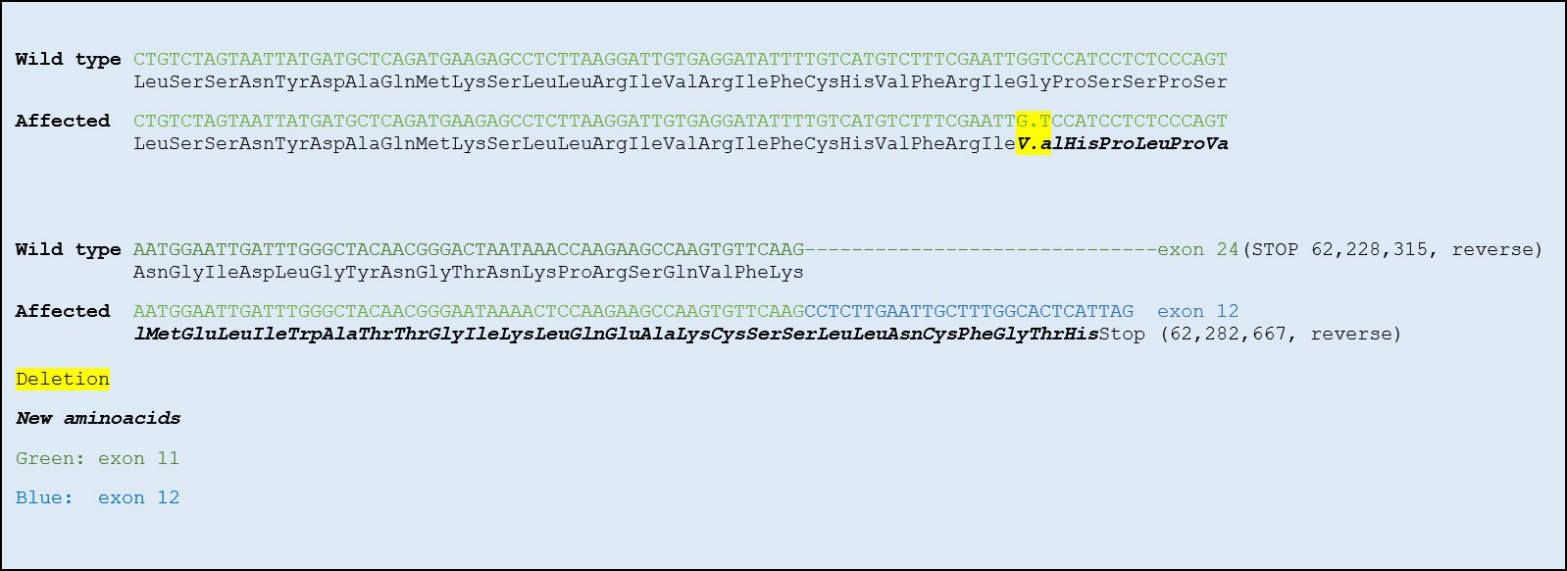
DNA-sequence and primary protein structure of exon 11 of the HACE1-gene in reference genome CanFam3.1 versus NEB ataxia case. The guanine-deletion cause a frameshift, which disrupts the reading frame and primary structure of the protein, including a premature stop codon.

**Fig 6:**
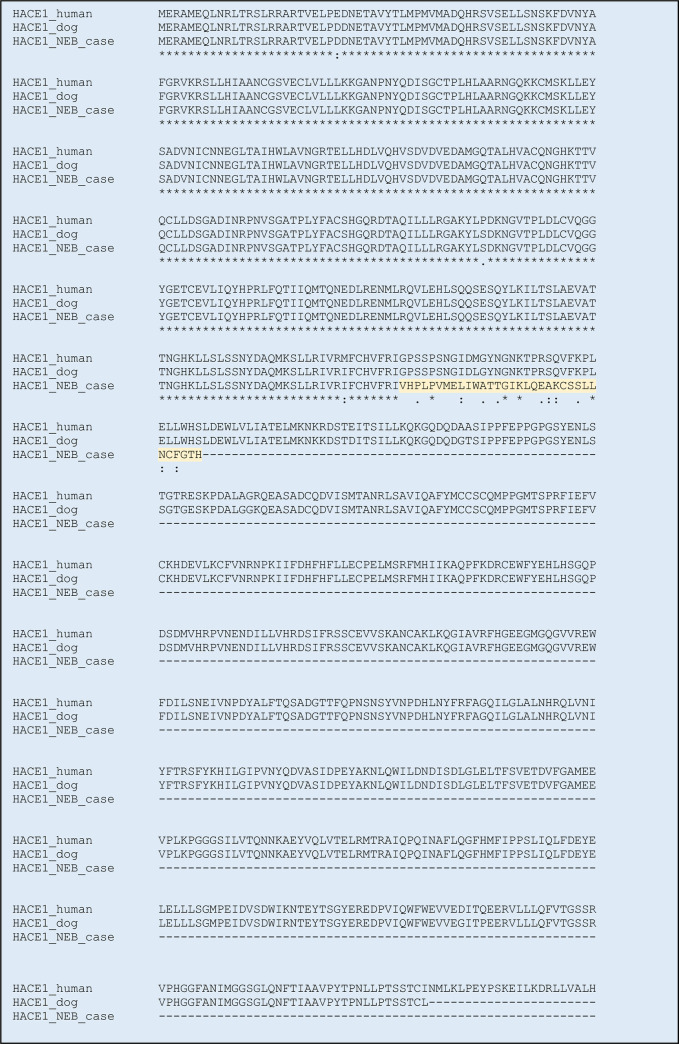
Full primary protein structure of the HACE1 in human, dogs (CanFam3.1) and NEB ataxia case, respectively. The abnormal protein contains 366 of 877 amino acids (511 amino acids too short), and the last 33 amino acids are erroneous.

**Fig 7:**
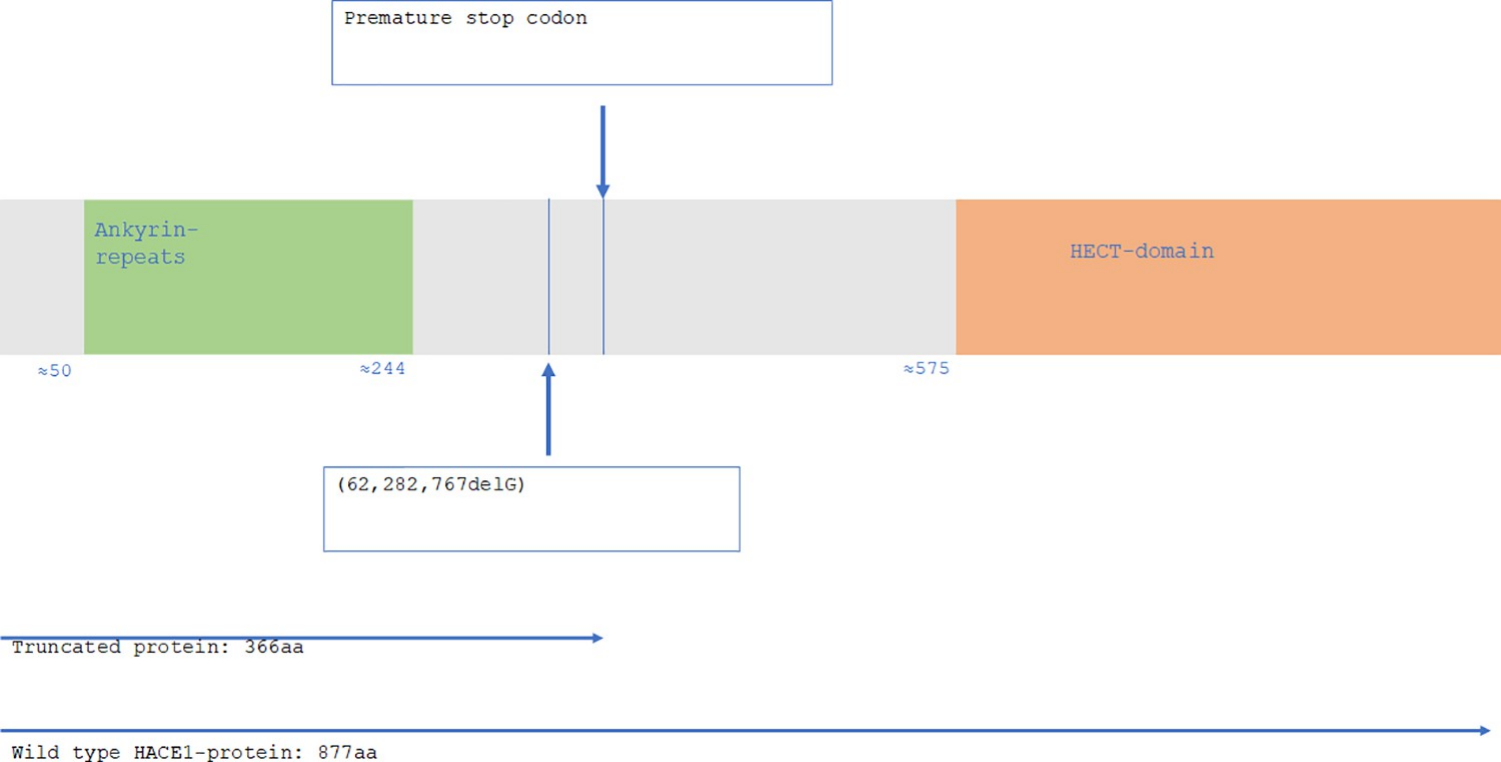
HACE1 domain structure. The domain structure of HACE1 with ankyrin-repeats (green) and HECT-domain (orange). NEB ataxia deletion and premature stop codon indicated with arrows.

HACE1 consists of 6 N-terminal ankyrin-repeats and a C-terminal HECT-domain. The guanine-deletion (62,282,767delG) identified in *HACE1* of the NEB ataxia-cases disrupts the reading frame and alters the primary structure of the protein. As a result, the protein is both erroneous and too short, due to a premature stop codon (Figs 5 and 6). The mutation causes loss of the entire, highly conserved [20], HECT-domain (Fig 7), which has been shown to entail loss of ubiquitination function [21]. The truncated protein encoded by the mutated *HACE1*-gene found in the NEB cases is presumably devoid of catalytic activity.

## Discussion

Through whole genome sequencing and subsequent variant calling we detected a novel 1 bp deletion in exon 11 of *HACE1*, in ataxic NEB. The mutation causes a frameshift, a premature stop codon in exon 12 and predicted loss of the entire HECT-domain of the HACE1 protein, which likely entails loss of the protein ubiquitination function. All cases (n = 8) were homozygote for the mutation and all parents of affected puppies (n = 10) were heterozygote. The mutation was not discovered in 308 dogs from 101 other breeds. Our finding presents a novel candidate gene for “non-syndromic ataxia” in dogs and humans.

HACE1 is an E3 ubiquitin ligase, which consist of an ankyrin repeat and a HECT domain. It facilitates the transfer of ubiquitin from E2 ubiquitin conjugating enzymes to tagged substrate proteins. HACE1 is therefore important in the degradation of small proteins, like RAC1 (Ras-related C3 botulinum toxin substrate 1). The ankyrin repeats are thought to be responsible for the protein recognition, whereas the HECT-domain is responsible for the ubiquitination process [22]. The protein is also a likely tumour suppressor [23].

Mutations in *HACE1* have been described in humans with a recessive neurodevelopmental syndrome called spastic paraplegia and psychomotor retardation with or without seizures (SPPRS) [21, 24–27] and *HACE1* mutations in cancer samples have been associated with uncontrolled cell proliferation [28]. Recently, *HACE1* mutations were identified in a patient with severe psychomotor- and mental retardation, diffuse cortical atrophy and 3-methylglutaconic aciduria [29]. Most of the mutations associated with SPPRS create premature stop codons and loss of the HECT-domain, which negatively affects the proteins ubiquitination function [22]. In cancer development, mutations in the ankyrin repeats and mid-section of the gene may be of equal or superior importance [28]. To the authors’ knowledge, *HACE1* has not previously been implicated in ataxia in humans or dogs.

*Hace1* is expressed throughout the adult mouse brain [24] and is important for embryonic development in *Xenopus laevis*, possibly through regulation of RAC1 activity [30]. Western blot analysis on brain tissue from *Hace1* knockout mice has shown that one of the downstream effects of HACE1 deficiency is increased levels of RAC1, and secondary increased levels of cyclin D1 and ROS [24]. *Rac1* is expressed diffusely in the murine brain during development [31] and is important in development of the nervous system [32].

Torpedoes in the Purkinje cell axons, like we observed in the NEB-cases, are reported in a wide range of diseases, such as spinocerebellar ataxias in humans [33] and abiotrophies in several dog breeds [34]. Additionally, torpedoes has been reported from transgenic ataxic mice expressing constitutive active Rac1 in Purkinje cells [35]. Thus, although further investigations are warranted, elevated levels of RAC1 may be part of the pathomechanism for the changes observed in ataxic NEB puppies. Interestingly, compared to other neurons, Purkinje cells remain viable for a longer time following axonal damage, and axotomy does not cause chromatolysis in the Purkinje cell soma by itself, possibly because the cells receive growth factor support locally from the Purkinje cell layer via axonal collaterals [36]. This peculiarity could explain the apparent lack of prominent changes in the Purkinje cell soma at this stage in the NEB-cases, despite the widespread changes in the Purkinje cell axons.

There are important clinical and pathological differences between our NEB ataxia cases and previously described human neurological disease caused by mutations in *HACE1*. The human neurological disease caused by mutations in *HACE1*, SPPRS, is typically characterized by hypotonia, progressive spasticity of the lower limbs, global developmental delay, mental retardation, speech difficulty, and seizures, as well as various congenital abnormalities [21, 24–26]. Magnetic resonance imaging findings from SPPRS patient typically include hypoplasia of the corpus callosum, a reduced white matter/grey matter-ratio, enlarged ventricles and cerebral atrophy [21, 24–26].

In the studied NEB cases, ataxia was the predominant clinical sign. The affected puppies had a broad-based kyphotic pelvic limb stance, a hanging tail that is atypical for the breed and delayed or absent postural reactions. No other obvious abnormalities were observed on clinical and neurological examinations: cranial nerves and spinal reflexes were unremarkable, the puppies were all afebrile and hematology and serum chemistry results displayed no abnormalities, except for one dog with an increased CRP value. CSF results were unremarkable, as was the CT-studies and the urine analysis. None of the owners reported seizures. No macroscopical changes were observed in post-mortem examinations. Post-mortem morphology revealed axonal swellings in the cerebellum and vacuoles in the brain stem.

Interestingly, the predominant neurological signs we observed in the NEB cases corresponds well to the localization of the observed pathological changes, which were in the cerebellum and brain stem. The clinical signs seen in SPPRS patients are different and involves several organs, and the pathological neurological changes are mainly located in the cerebrum. Our results show that mutations in *HACE1* can affect other regions of the brain than previously described and subsequently cause a wider spectrum of neurological diseases. This makes *HACE1* a novel candidate gene for ataxia and cerebellar dysfunction. Since *HACE1* mutations are associated with a syndrome in human (SPPRS) [21] and we found no indication (post-mortem and CT) that other organs were affected in the NEB cases, we have called this a non-syndromic form of ataxia. We have, however, not been able to evaluate some symptoms associated with SPPRS (speech delay, hearing loss, obesity, seizures) in the dog model, partly because the dogs were euthanized at a low age.

Further investigations are warranted to better understand why most of the mutations previously described in humans seem to affect the cerebrum, whilst the mutation discovered in ataxic NEB appear to primarily affect the cerebellum and brain stem. One possible explanation is that the NEB *HACE1* mutation may alter the protein in a different manner than previously described mutations. The nature and position of the NEB *HACE1* mutation indicates that the effect on protein structure should be similar to that of SPPRS patients, as both entail loss of the entire HECT-domain and predicted complete loss of function. However, much is still unknown about the pathophysiology of disease caused by *HACE1* mutations [29], as well as the function of all parts of the HACE1 protein, especially the middle domains [28].

Another possible explanation could be that the NEB cases and SPPRS patients are evaluated at different stages of disease development. Because the NEB cases were euthanized at a young age for animal welfare reasons, we cannot rule out that they might have developed observable lesions in the cerebrum with compatible neurological signs at a later stage. However, neurological evaluations of SPPRS patients have been conducted at different ages and at no point in development does the findings in humans appear identical to the findings in the elkhounds. If the development of the symptoms had been similar in human and dogs, we would expect at history of ataxia as a predominant symptom at a young age in the SPPRS patients, which is not the case.

Additionally, there could be differences in the expression pattern of *HACE1* in dog versus human, that contribute to the different biological effect of *HACE1* mutations in the two species. We know that dogs have a poorly developed pyramidal system compared to primates. The relative importance of the pyramidal system in the two species could potentially influence the differences in expression patterns in dog versus primate.

There is a need for more information about the pathophysiology of disease caused by mutations in *HACE1* to better understand the role of HACE1 in a wide spectrum of diseases. Our results have identified a new phenotype associated with *HACE1* and provide a novel candidate gene for non-syndromic cerebellar ataxia. Several of the genes that harbour mutations that have been found to cause ataxia in dogs, are also associated with ataxia in humans. We therefore suggest that the spontaneous *HACE1* mutation in dog may serve as an attractive model for ataxia in human.

The perfect association between the identified mutation, combined with the predicted significant molecular effect, strongly suggest that we have found the causative mutation for Norwegian elkhound black ataxia. DNA-testing can be utilized to prevent matings between two carriers and the birth of affected puppies, and possibly to eradicate the condition from the population over time. Our result provides a new candidate gene for ataxia in other dog breeds and species, including human.

## Materials and methods

### Dogs

Initially, three affected NEB puppies were detected in a litter of six (Litter 1, born 2016). The puppies showed signs of ataxia with weakness in the hind limbs from 3–4 weeks of age. The puppies were presented to the Norwegian University of Life Sciences, Faculty of Veterinary Medicine, for a thorough clinical examination. Due to the severity of clinical signs and poor prognosis, the puppies were euthanized for animal welfare reasons, at the owner’s request. Relevant tissues from the cerebrum, cerebellum, brain stem, spinal cord and peripheral nerves were collected for morphology post-mortem. Tissue samples were also snap-frozen in liquid nitrogen immediately after death.

Another affected puppy was reported from a second litter in 2018 (Litter 2, 6 puppies in total). The puppy had already been euthanized by the local veterinarian and had been frozen (-20°C) immediately after death. A third litter with one affected puppy with the same clinical signs as the previous four, were then discovered in a litter of two (Litter 3, born 2018). Another affected puppy was discovered in the summer of 2020 (Litter 4, 2 puppies in total). Eventually a litter with two affected puppies was discovered in May 2021 (Litter 5, 2 puppies in total). An overview of litters, dogs and phenotypes can be found in Table 2.

**Table 2:** Overview of litters, dogs, and phenotypes.

Dogs	Sex	Litter	Year of birth	Phenotype	Neurological examination	Autopsy	Whole genome sequenced
Sire	Male	Litter 1		Unaffected			X
Dam	Female	Litter 1		Unaffected			X
Puppy 1	Male	Litter 1	2016	Affected	X	X	X
Puppy 2	Female	Litter 1	2016	Affected	X	X	
Puppy 3	Female	Litter 1	2016	Affected	X	X	
Puppy 4	Male	Litter 1	2016	Unaffected			X
Puppy 5	Female	Litter 1	2016	Unaffected			
Puppy 6	Female	Litter 1	2016	Unaffected			
Sire	Male	Litter 2		Unaffected			X
Dam	Female	Litter 2		Unaffected			X
Puppy 7	Female	Litter 2	2018	Affected		X	X
Puppy 8	Female	Litter 2	2018	Unaffected			X
Puppy 9	Male	Litter 2	2018	Unaffected			
Puppy 10	Female	Litter 2	2018	Unaffected			
Puppy 11	Female	Litter 2	2018	Unaffected			
Puppy 12	Female	Litter 2	2018	Unaffected			
Sire	Male	Litter 3		Unaffected			X
Dam	Female	Litter 3		Unaffected			X
Puppy 13	Male	Litter 3	2018	Affected	X	X	X
Puppy 14	Female	Litter 3	2018	Unaffected			X
Sire	Male	Litter 4		Unaffected			
Dam	Female	Litter 4		Unaffected			
Puppy 15	Female	Litter 4	2020	Affected	X	X	
Puppy 16	Male	Litter 4	2020	Unaffected			
Sire	Male	Litter 5		Unaffected			
Dam (puppy 5)	Female	Litter 5		Unaffected			
Puppy 17	Male	Litter 5	2021	Affected		X	
Puppy 18	Female	Litter 5	2021	Affected		X	

DNA-samples were collected from all dogs in the 5 litters and all the involved parents. The five affected puppies in litters 1, 3 and 4 were subjected to clinical and neurological evaluations, euthanized with an intravenous barbiturate injection for animal welfare reasons and subjected to post-mortem pathomorphological evaluations.

### Clinical characterization of affected dogs

Five ataxic puppies (from litter 1, 3 and 4) with a history of progressive neurological signs were clinically and neurologically examined in the Small Animal Teaching Hospital at the Norwegian University of Life Sciences, Faculty of Veterinary Medicine. The puppies were between 8 and 12 weeks at examination. Examinations followed standardized protocols of the hospital and were performed by, or personally supervised by, a board-certified neurologist (KHJ). A complete hematological profile including the number of blood cells and measurements of other hematological parameters(WBC, RBC, HGB, HCT, MCV, MCHC, RDW, PLT) in addition to an automated five-part leukocyte differential count (neutrophils, lymphocytes, monocytes, eosinophils and basophils) were analyzed in EDTA stabilized sample tubes from each puppy using an Advia 2120^®^ Hematology Analyzer (Siemens Healthcare GmbH). The automated leukocyte differential count and blood cell morphology were also evaluated microscopically by an experienced laboratory technician. In addition, serum chemistry (AST, ALT, AP, CK, amylase, lipase, TP, albumin, globulin, urea, creatinine, bile acids, total bilirubin, cholesterol, glucose, inorganic phosphorous, calcium, sodium, potassium, chloride and CRP) were analyzed on ADVIA 1800^®^ Clinical Chemistry System (Siemens Healthcare GmbH). Cerebrospinal fluid was sampled from the cerebellomedullary cistern from the five dogs under general anesthesia and was investigated regarding cell count, cytology, and protein concentration. In addition, three dogs were sedated and underwent a computed tomography (CT)-examination of the brain and spine, using a Lighspeed, 4 slice GE scanner, pre-and post-intravenous injection of contrast (Omnipaque, 300mg Iodine/ml, GE healthcare). A urine sample obtained by cystocentesis was examined (macroscopy, chemical analysis, specific gravity, sediment) from one puppy.

### Morphological assessment

Samples for light microscopy were fixed in formalin and paraffin-embedded. Sections were stained with the following methods: Hematoxylin and eosin (HE), Luxol fast blue/cresyl violet, Periodic acid-Schiff (PAS) and Bodian stain. Additionally, immunohistochemistry with antibodies against neurofilament and glial fibrillary acidic protein (GFAP) and immunofluorescence with antibodies against neurofilament and myelin basic protein were performed on sections from cerebellum.

For electron microscopy, thin slices of the nervous tissue were fixed in 2.5% glutaraldehyde in Sorensen’s phosphate buffer (0.1 M, pH 7.4), dehydrated in an ascending acetone series and embedded in epon. Semi thin (500 nm) sections were stained with toluidine blue. Ultra thin (70 nm) sections were contrasted with uranyl acetate and lead citrate, and studied in a FEI Morgagni 268 transmission electron microscope (FEI, Hillsboro, Oregon, United States) equipped with an Olympus Veleta CCD camera.

### Preparation of DNA

DNA was extracted from EDTA-blood and buccal swabs (Performagene®). In addition to the NEB-cases and their relatives, DNA was also extracted from blood samples from healthy dogs, collected for routine hematological analysis, with owners’ consent. DNA from blood samples was extracted using an E.Z.N.A blood DNA kit (Omega Bio-Tek, Norcross, GA, USA). DNA from buccal swabs was extracted following the manufacturer’s recommendations. DNA was stored at –20°C.

### Whole genome sequencing

12 DNA samples, comprising both parents, one affected puppy and one healthy littermate from litters 1–3 were prepared for sequencing (Fig 1). The samples were sequenced at the Norwegian Sequencing centre (NSC) using Illumina technology on a HiseqX. Libraries were constructed at NSC using TruSeq^TM^ PCR-free prep and 150bp PE sequencing with approximately 24X coverage. The sequences are available at the European Nucleotide Archive with accession number PRJEB45384 ()

### Variant calling

Sequences were uploaded to the portal of the Norwegian e-Infrastructure for Life Sciences (NeLS) [37] and bioinformatic work were performed using the national Abel gene cluster at the University of Oslo.

Alignment of the sequences was performed with BWA-mem [38] to the reference genome (CanFam 3.1). The GATK4 recommended best practice (Broad institute) for variant calling was followed.

12 dogs were sequenced (whole genome) and the sequences were combined with WGS-sequences from three salukis from an inhouse repository of projects. After alignment, haplotyping and genotyping of each sequence, variant calling was performed on the combined gvcf-file. Variant calling was performed using “selectvariants” with appropriate JEXL-programming using a recessive model.

Variants were selected where the six parents in all three litters (1–3) were heterozygotes, affected offspring (1+1+1) were homozygote variant, sequenced healthy littermates (1+1+1) were not homozygous variant and the three salukis were homozygote reference.

### Additional genotyping

Dogs from litter 4 and 5, which were detected after the initial sequencing of litter 1–3, were genotyped. The deletion segregated as in litter 1–3 and confirmed the initial findings.

We also genotyped 24 unrelated samples from Norwegian elkhound grey and 308 dogs from 101 different breeds without detecting the deletion. Primers used were forward: CCTGTCATGGTAGGAATTTGC and reverse: TGAGGTTTCTGACTAATGAAAGCA (sequence); and forward: GAGCCTCTTAAGGATTGTGAGGA and reverse: AGAGGCTTGAACACTTGGCT (fragment). Fragment cycle conditions were as follows: 95°C for 2 minutes and 30 seconds; 35 cycles of 95°C for 30 seconds, 58°C for 40 seconds, and 72°C for 50 seconds; and 72°C for 5 minutes and 30 seconds.

### Ethics statement

All examination and samples of involved cases were performed as part of the necessary diagnostic work-out by board-certified veterinarians with owner’s consent according to ethical guidelines of the Norwegian University of Life Sciences (NMBU). The Saliva-swabs and blood samples were collected by certified veterinarians at Norwegian University of Life Sciences (NMBU) or in private veterinary clinics during routine clinical work or genotyping purposes with owner’s consent. All investigations were in agreement with the provisions enforced by the Norwegian Animal Research Authority and the Norwegian Regulation on Animal Experimentation.
